# Interaction between central and peripheral vision: Influence of distance and spatial frequencies

**DOI:** 10.1167/jov.24.1.3

**Published:** 2024-01-08

**Authors:** Cynthia Faurite, Eva Aprile, Louise Kauffmann, Martial Mermillod, Mathilde Gallice, Christophe Chiquet, Benoit R. Cottereau, Carole Peyrin

**Affiliations:** 1 Université Grenoble Alpes, Univ. Savoie Mont Blanc, Grenoble, France; 2 Department of Ophthalmology, Grenoble Alpes University Hospital, Grenoble, France; 3 Centre de Recherche Cerveau et Cognition, Université Toulouse III–Paul Sabatier, Toulouse, France; 4 Centre National de la Recherche Scientifique, Toulouse, France

**Keywords:** scene perception, object recognition, congruence effect, predictive brain, retinotopy

## Abstract

Visual scene perception is based on reciprocal interactions between central and peripheral information. Such interactions are commonly investigated through the semantic congruence effect, which usually reveals a congruence effect of central vision on peripheral vision as strong as the reverse. The aim of the present study was to further investigate the mechanisms underlying central-peripheral visual interactions using a central-peripheral congruence paradigm through three behavioral experiments. We presented simultaneously a central and a peripheral stimulus, that could be either semantically congruent or incongruent. To assess the congruence effect of central vision on peripheral vision, participants had to categorize the peripheral target stimulus while ignoring the central distractor stimulus. To assess the congruence effect of the peripheral vision on central vision, they had to categorize the central target stimulus while ignoring the peripheral distractor stimulus. Experiment 1 revealed that the physical distance between central and peripheral stimuli influences central-peripheral visual interactions: Congruence effect of central vision is stronger when the distance between the target and the distractor is the shortest. Experiments 2 and 3 revealed that the spatial frequency content of distractors also influence central-peripheral interactions: Congruence effect of central vision is observed only when the distractor contained high spatial frequencies while congruence effect of peripheral vision is observed only when the distractor contained low spatial frequencies. These results raise the question of how these influences are exerted (bottom-up vs. top-down) and are discussed based on the retinocortical properties of the visual system and the predictive brain hypothesis.

## Introduction

In the real-world visual environment, objects are rarely perceived in isolation but within a rich and complex scene context. Different objects in a scene can more or less frequently co-occur and maintain typical spatial relation (Bar & Ullman, 1996), so that observers can extract regularities and acquire knowledge about the objects found in a particular context. Based on this knowledge, the recognition of objects in a part of a scene could facilitate the recognition of adjacent objects. This hypothesis has in fact been the topic of many studies that have investigated the visual interactions between objects and scene context through the semantic congruence effect (see among others, Bar & Ullman, 1996; Biederman, 1972; Boucart, Moroni, Szaffarczyk, & Tran, 2013; Brandman & Peelen, 2017; Brandman & Peelen, 2019; Davenport, 2007; Davenport & Potter, 2004; Joubert, Fize, Rousselet, & Fabre-Thorp, 2008; Joubert, Rousselet, Fize, & Fabre-Thorpe, 2007; Katti, Peelen, & Arun, 2017; Lauer, Schmidt, & Vô, 2021; Mudrik, Lamy, & Deouell, 2010; Mudrik, Shalgi, Lamy, & Deouell, 2014; Palmer, 1975; Rémy et al., 2013; Roux-Sibilon et al., 2019; Spaak, Peelen, & de Lange, 2022; Truman & Mudrik, 2018). Typically, detection, categorization or identification of objects is better within a congruent scene context (i.e., consistent with knowledge about the visual environment) than in an incongruent scene context. But the reverse is also true. The categorization of a scene context can also be influenced by the congruence of an object in the scene. Therefore the congruence effect is considered as proof of interaction and integration between objects and scene context. Knowledge about objects and scene contexts that tend to co-occur influences perception (Davenport, 2007).

Under real-life conditions, observers most often foveate different objects in a coherent/congruent scene context. In other words, central vision is more suited to the perception of each object and peripheral vision to the perception of the remaining part of the scene. Thus congruence effects between objects and scene context raise the question of the interaction between central and peripheral vision. This question was only partially addressed in previous studies on the congruence effects between objects and context, since the distinction between central and peripheral vision was not their research focus, and these have generally neither controlled the position of the object in the scene context, nor the size of the objects on the retina (see however, Roux-Sibilon et al., 2019). Two recent studies (Lukavský, 2019; Trouilloud et al., 2022) directly tackled this issue using stimuli composed of a central disk and a peripheral ring either congruent (belonging to the same scene image) or incongruent (belonging to two different scene images from different categories) and presented briefly (less than 100 ms). Participants had to ignore the peripheral ring when categorizing the central disk in one condition and to ignore the central disk when categorizing the peripheral ring in another one. Results showed that whatever the location of the information to ignore (central or peripheral), the categorization of the information to attend (peripheral or central, respectively) was better for congruent than incongruent stimuli. These studies suggest that information to be ignored is automatically processed and integrated to the information to be categorized. Although these results may imply that central and peripheral vision strongly interact during scene categorization, the mechanisms underlying these interactions are poorly understood.

Trouilloud et al. (2022) postulated that the interaction between central and peripheral vision rely on top-down processes. This hypothesis is based on a predictive conception of the brain according to which visual perception depends on the sensory inputs, but also on expectations about them (Bar, 2007; Clark, 2013; de Lange, Heilbron, & Kok, 2018; Friston, 2005; Rao & Ballard, 1999; Trapp & Bar, 2015). In the framework of scene and object recognition (Bar, 2003; Bar, 2007; Kveraga, Boshyan, & Bar, 2007; Kauffmann, Chauvin, Pichat, & Peyrin, 2015; Oliva & Torralba, 2006; Peyrin et al., 2010), the rapid extraction and conduction of low spatial frequencies (LSF) from the retina to the brain would allow a global and rudimentary representation of the scene (known as the gist) that could be associated with prior knowledge about regularities in the visual environment (known as contextual associations). Knowledge could then be used to generate predictions about objects and details that compose the scene. These predictions could then guide the subsequent and slower processing of information contained in high spatial frequencies (HSF). This brain mechanism would be however constrained by the anatomical and functional properties of the retinal cells (Curcio & Allen, 1990; Curcio, Sloan, Kalina, & Hendrickson, 1990; Wässle, Grünert, Röhrenbeck, & Boycott, 1990): Midget ganglion cells, selective to HSF, are numerous in the fovea and disappear with retinal eccentricity. Parasol ganglion cells, selective to LSF, can be found in the fovea but their density increases with retinal eccentricity. This functional organization is preserved at the level of the visual cortex (Musel et al., 2013; Henriksson, Nurminen, Hyvärinen, & Vanni, 2008; Ramanoël, Kauffmann, Cousin, Dojat, & Peyrin, 2015; Sasaki et al., 2001; Singh, Smith, & Greenlee, 2000). For example, using a categorization task of filtered scenes, Musel et al. (2013) observed that the categorization of LSF scenes elicited the selective activation of the anterior part of the occipital lobe dedicated to peripheral vision, in relation to the higher density of LSF-selective ganglion cells in the retina, whereas the categorization of HSF scenes elicited the selective activation of the posterior occipital lobes dedicated to central vision in relation to the higher density of HSF-selective ganglion cells in the retina. Therefore, as LSF are mainly extracted in peripheral vision, Trouilloud et al. (2022) expected a stronger influence from peripheral to central vision than the reverse. However, similarly to Lukavský (2019), the authors did not observe such prioritization of peripheral vision in congruence effects suggesting that congruence effects do not necessarily rely on the rapid processing of LSF in peripheral vision. Information from the central and the peripheral vision, even those to be ignored, only accumulates gradually until the decision on the category.

However, Trouilloud et al. (2022) wondered about a potential methodological bias in their experiment. Indeed, in order to ensure a central versus peripheral presentation of the stimuli, participants in their experiment were instructed to fixate a central point (monitored by eye movement recordings) throughout the experiment, potentially inducing an unexpected selective attention on central vision (i.e., the preferential processing of central vision with some concomitant decrease in processing of remaining information) even when this part of the visual field has to be ignored. Given that scene perception is known to involve distributed attention over central and peripheral vision (Brand & Johnson, 2018), this experimental instruction could thus have excessively increased the influence of central vision on peripheral vision relative to natural viewing conditions, or alternatively hindered the influence of peripheral vision on central vision. These authors also wondered about their original hypothesis. As previously mentioned, parasol ganglion cells are also present in the fovea (Kolb, Nelson, Ahnelt, Ortuño-Lizarán, & Cuenca, 2020) and LSF can be extracted in central vision. As evidence, detection of small sine-wave gratings and categorization of small filtered scenes are faster in LSF than HSF (Breitmeyer, 1975; Kauffmann et al., 2017). In addition, studies using small hybrid images as stimuli also showed that categorization of HSF scenes is better when superimposed on a congruent LSF scene than on an incongruent scene, suggesting an efficient processing of LSF in central vision (Kauffmann, Bourgin, Guyader, & Peyrin, 2015; Kauffmann et al., 2017; Mu & Li, 2013). Therefore, top-down predictive processes based on the rapid processing of LSF may also originate from central vision and could explain congruence effects as strong from central vision as from peripheral vision. We also considered the hypothesis that congruence effects could in addition rely on the bottom-up retinotopic organization of spatial frequency processing from retina to visual cortex (Musel et al., 2013; Henriksson et al., 2008; Ramanoël et al., 2015; Sasaki et al., 2001; Singh et al., 2000): HSF information mainly activates neural populations receiving inputs from central vision, whereas LSF information mainly activates neural populations processing peripheral vision.

The aim of the present study is to further investigate the interaction between central and peripheral vision through congruence effects by considering the major methodological bias mentioned above (Experiments 1) and also addressing theoretical hypotheses (Experiments 2 and 3). For this purpose, we presented simultaneously two stimuli of different retinal eccentricities, one central and one peripheral, that could be either congruent (belonging to the same scene image) or incongruent (belonging to two different scene images from different categories). In one experimental session assessing the congruence effect of central vision on peripheral vision, participants had to categorize the peripheral target stimulus (as an indoor or an outdoor scene) while ignoring the central distractor stimulus (Peripheral target condition). In another session assessing the congruence effect of the peripheral vision on central vision, they had to categorize the central target stimulus while ignoring the peripheral distractor stimulus (Central target condition). Experiment 1 aimed at addressing the potential bias induced by a central fixation instruction throughout the experiment. In this experiment, we used two different central stimuli of different eccentricities. One stimulus was a small central disk superimposed on the central fixation, as in previous studies (Lukavský, 2019; Trouilloud et al., 2022). The other one was a central ring adjacent to the central disk and thus slightly distant from the central fixation point. In this condition, we expected that the processing of the semantic information in central vision was not biased by the selective attention on central fixation. Therefore, the congruence effect from the central to the peripheral stimulus should be lower when the central stimulus is distant than superimposed on the central fixation. In parallel with Experiment 1, Experiments 2 and 3 were theoretically motivated and specifically addressed whether the congruence effects between central and peripheral vision rely on the rapid processing of LSF. To test this hypothesis, we manipulated the spatial frequency content of distractors (either filtered in LSF or HSF). If congruence effects result from the rapid extraction of a LSF content that triggers top-down predictive processes, we expected a stronger congruence effect from LSF than HSF distractors irrespective of their location in the visual field. Congruence effects may also depend on the bottom-up retinotopic sensitivity of the visual system for processing spatial frequencies. In such a case, we should observe an interaction between the congruence of stimuli, the location of the target and the spatial frequency content of the distractor: A central HSF distractor should induce a greater congruence effect than a central LSF distractor, while a peripheral LSF distractor should induce greater congruence effect than a HSF peripheral distractor. We also considered the hypothesis that these two mechanisms act concomitantly. In Experiment 3, we investigated whether semantically congruent distractors caused a facilitation or semantically incongruent distractors caused an interference on categorization by including a neutral condition in which the distractor was a 1/f noise without semantic content.

## Experiment 1

### Method

#### Participants

Twenty undergraduate students of Psychology from University Grenoble Alpes (14 women; *M* ± *SD* = 18.95 ± 0.95 years) participated in the experiment. The sample size was chosen by realizing a power analysis based on the effect sizes of Trouilloud et al. (2022). Three effect sizes were examined to achieve power of 0.99 at an alpha level of 0.05. The effect size of the congruence effect irrespective of the position of the distractor (*d*z = 1.685) provided a sample size of 9 participants. The effect size of the congruence effect from peripheral to central vision (*d*z = 1.296) provided a sample size of 14 participants. And finally, the effect size of the congruence effect from central to peripheral vision (*d*z = 1.08) provided a sample size of 18 participants. Therefore, we fixed a sample size of 18 participants in order to ensure a congruence effect in both distractor position conditions to investigate the influence of the new manipulated factors. We recruited 20 participants, assuming that a few of them would not perform the task correctly. Before the experiment, participants performed a visual acuity test, the FrACT, Freiburg Visual Acuity Test (Bach, 1996). All participants were right-handed and had normal or corrected-to-normal vision. Participants with a logMAR (logarithm of the Minimum Angle of Resolution) higher than 0.1 were excluded. At the end of the experiment, they received course credits for their participation. All participants gave their informed written consent before participation. This study is approved by the local ethics committee of University Grenoble Alpes (CERGA, IRB00010290).

#### Stimuli

Stimuli were constructed from 50 scene images belonging to two different categories (indoor and outdoor) selected from a photo sharing website (Pixabay, https://pixabay.com). Half of the photographs represented indoor scenes (e.g., bedroom, bathroom, kitchen) and the other half represented outdoor scenes (e.g., beach, mountain, cityscape). We ensured that a semantically congruent object of the scene category (e.g., a kitchen utensil in an indoor scene, a boat in an outdoor scene) was present in a central square part of the image.

**Figure 1.**
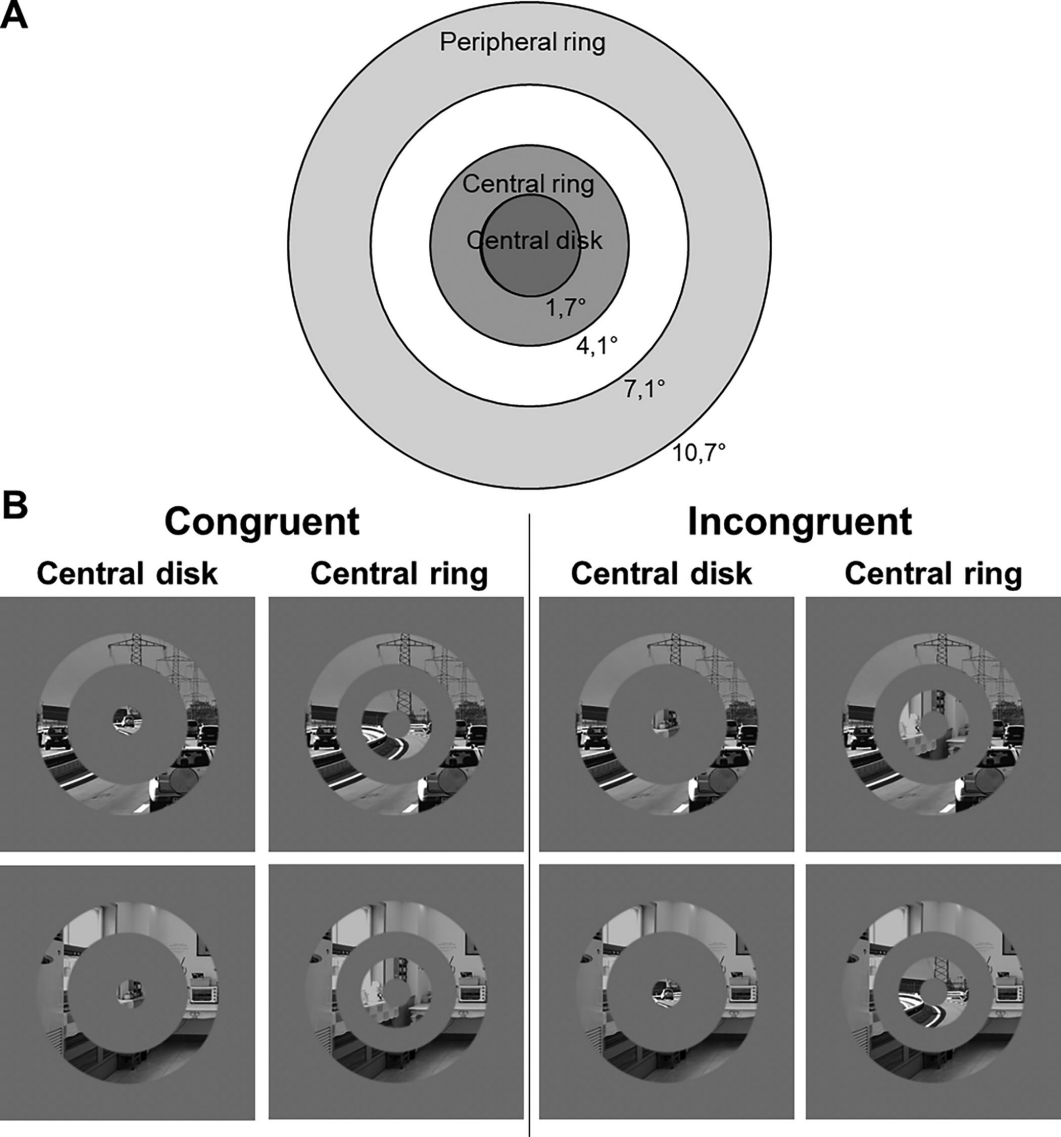
(**A**) Illustration of the eccentricities (in degree of visual angle) of the stimuli's inner and outer edges. (**B**) Example of stimuli displayed in Experiment 1. The central stimulus (central disk or central ring) and the peripheral stimulus were presented simultaneously. Stimuli could be either congruent (a central outdoor scene and the same peripheral outdoor scene or a central indoor scene and the same peripheral outdoor scene) or incongruent (a central outdoor scene and a peripheral indoor scene or a central indoor scene and a peripheral outdoor scene).

Stimuli were built using MATLAB (MathWorks Inc., Sherborn, MA, USA) based on the procedure used in Trouilloud et al. (2022). All images were converted to 256 gray levels by averaging the values from the three color channels at each pixel. We also normalized them to a mean luminance of 128 pixel intensity on a 256 gray level scale, and a mean root mean square contrast of 51 on a scale of 256 gray levels. We then extracted stimuli of different eccentricities (Figure 1a) considering (a) that central vision is usually considered to extend from 0° to 5° eccentricity, including both foveal and parafoveal vision (Loschky, Szaffarczyk, Beugnet, Young, & Boucart, 2019), (b) the cortical magnification factor (i.e. stimuli activate the same cortical surface on V1 using an empirically derived equation from retinotopic measurement; Geuzebroek, & van den Berg, 2018; Trouilloud et al., 2020; Trouilloud et al., 2022; Wu, Yan, Zhang, Jin, & Guo, 2012), and (c) a viewing distance of 70 cm for our participants. Thus central stimuli were a small disk of 1.7° radius (83 pixels), and a ring adjacent to the disk for which the inner and outer edges were respectively fixed at 1.7° and 4.1° of visual angle (i.e., 83 and 205 pixels) from the central position of the photographs. The peripheral stimulus was a ring just beyond the parafovea for which the inner and outer edges were respectively fixed at 7.1° and 10.7° of visual angle (i.e., 355 and 535 pixels) from the central position of the photographs. Edges of stimuli were smoothed to avoid local contrast differences between conditions and artificially introducing high spatial frequency components in stimuli. Stimuli were constructed by multiplying the original scene image with binary masks of the ring and the central disk. These binary masks were spatially filtered by a Gaussian with a standard deviation of 5 prior to multiplication with the scene. In total we had 50 central disks, 50 central rings, and 50 peripheral rings.

During the experiment, a central stimulus (either the central disk or the central ring) and a peripheral stimulus (the peripheral ring) were simultaneously presented (Figure 1b). This compound stimulus could be either congruent (e.g., an indoor scene in the central stimulus with the same indoor scene in the peripheral stimulus) or incongruent (e.g., an indoor scene in the central stimulus with an outdoor scene in the peripheral stimulus). It should be noted that we used the same image for central and peripheral stimuli in the congruent condition in order (1) to avoid physical implausibility (e.g., differences in object size contained in the two stimuli) that could be interpreted as a semantic incongruence by the visual system, but also (2) to limit physical dissimilarities. Indeed, Peyrin et al. (2021) demonstrated that physical dissimilarities between two semantically congruent central and peripheral scenes impaired the categorization of the central scene, and thus decreased congruence effects. As the aim of the present experiment was to compare the congruence effect of peripheral vision to the congruence effect of central vision, we expected to maximize congruence effect using the same scene image for central and peripheral stimuli in the congruent condition. In this way, the congruent condition is closer to the natural conditions of vision. Stimuli can be downloaded from https://osf.io/39gf6/.

#### Procedure

Stimuli were displayed using Psychtoolbox (Brainard, 1997; Kleiner et al., 2007) implemented by MATLAB R2019 (MathWorks, Natick, MA, USA) on a 30″ monitor DELL ULTRASHARP (60 Hz), with a visual resolution of 2560 × 1600 pixels in a darkened room. In order to respect the stimuli angular size, participants’ heads were placed on a chin rest at 70 cm from the screen. All participants performed four experimental sessions. In two experimental sessions, we assessed the influence of peripheral vision on central vision. Participants had to categorize the central target stimuli (indoor vs. outdoor categorization), while ignoring the peripheral distractor stimulus (central target/peripheral distractor task). The central distractor stimulus was the central disk in one session and the central ring in the other session. In the two other experimental sessions, we assessed the influence of central vision on peripheral vision. Participants had to categorize the peripheral target stimuli (indoor vs. outdoor categorization), while ignoring the central distractor stimulus (peripheral target/central distractor task). The central distractor stimulus was the central disk in one session and the central ring in the other session. The order of the four experimental sessions was counterbalanced between participants. For each session, a trial began with a central black fixation point of 0.8° of visual angle (20 pixels) presented for 500 ms on a gray background of 128 pixel intensity, immediately followed for 100 ms by a compound stimulus (either the central disk and peripheral ring or the central ring and the peripheral ring, depending on the experimental session) also displayed on a gray background of 128 pixel intensity. Finally, the trial ended with a gray screen of 128 pixel intensity for 1900 ms during which participants could respond. Participants were instructed to categorize the target stimulus (either the central or the peripheral stimulus) as an indoor or an outdoor scene as quickly and as correctly as possible by pressing the corresponding response key with the middle finger and forefinger of their right hand. They were also instructed to fixate the center of the screen during the whole experimental session. To ensure that, eye movements were recorded throughout the experiment. We used an Eyelink 1000 eye-tracker (SR Research) with a nominal spatial resolution of 0.01° of visual angle and a sampling rate of 1,000 Hz. For each participant, we recorded only the left eye using the “pupil-corneal reflection” mode. The Eyelink software detected saccades with a velocity threshold greater than 30°/s, an acceleration greater than 8000°/s2, and a saccadic displacement greater than 0.15°. Fixations were detected when no saccade was in progress and the pupil was visible. Blinks were detected during occlusion (partial or total) of the pupil. At the beginning of each experimental session, a calibration procedure was realized in which participants were asked to orient their gaze toward nine separate dots appearing sequentially in a 3 × 3 grid occupying the whole screen. A single-point calibration check in the center of the screen was performed every 10 trials and a new calibration was done if the error rose above 0.5°.

Each experimental session included 80 trials (20 congruent stimuli for the indoor category, 20 congruent stimuli for the outdoor category, and 40 incongruent stimuli). Half of the trials had a central disk stimulus, and the other half had a central ring stimulus. Thus, the whole experiment included 320 trials and lasted around 45 minutes. For each trial, we recorded the response accuracy and the response time in milliseconds (ms). Before starting each experimental session, participants achieved a training session of 20 trials with stimuli that were not included in the main experiment.

#### Data analysis

Data analyses were performed using the *lme4* package (Bates, Mächler, Bolker, & Walker, 2014) in R (R Core Team 2019). For the error rates (ERs) analysis, we performed a generalized linear mixed-effect model (binomial family). Participants’ incorrect responses were coded 1 and correct responses were coded 0. For the correct response times (RTs) analysis, we performed a linear mixed-effects model of the Congruence of the distractor (congruent vs. incongruent), the Distractor position (peripheral distractor vs. central distractor) and the Eccentricity of the central stimulus (central disk vs. central ring) in a 2 × 2 × 2 within-subjects design. For each model we included participants as a random factor. Mixed-effects models have the advantage to estimate and correct for outliers. We chose to keep participants who could be considered as outliers in our analyses in order to avoid the bias of removing inconvenient data just to fit our model. Doing so, we aimed at maintaining a variance that is close to the reality of our data. With the goal to generalize our results to other participants, we specified as random effects the intercepts for participants, along with participants-wise random slopes for the Congruence, Distractor position, Eccentricity of the central stimulus, and their mutual interactions. It should be noted that we did not include items as random effects because each item (i.e., each combination of central and peripheral stimuli) was not seen in each condition of the Congruence factor which was our main factor of interest. This however entails that any observed congruence effect or its interaction with other factors cannot be generalized to any other visual stimulus. We constructed parsimonious mixed models through the method of Bates, Kliegl, Vasishth, and Baayen (2015), allowing us to prevent convergence problems. The visual analysis of residual plots failed to reveal any violation of homoscedasticity and normality. We set the significant threshold of *p*-values at a standard *p* < 0.05 criteria and we estimated the effect sizes using Cohen's D_z_ (Lakens, 2013). Data from one participant who did not perform the four experimental sessions were removed from the analyses. We conducted the statistical analyses on 19 participants (13 women; 18.95 ± 0.97 years). Trials for which the starting fixation location was beyond 1.7° of eccentricity around the fixation point (corresponding to the radius of the central disk) or for which a saccade was initiated beyond 1.7° of eccentricity around the fixation point were discarded from the analyses. Therefore we removed 3.73% of the trials. To avoid increasing the risk of Type-1 error, we performed the minimum number of statistical analyses to test our hypotheses.

Regarding previous studies (Lukavský, 2019; Trouilloud et al., 2022), we expected to observe a main congruence effect irrespective of the Distractor position and of the Eccentricity of the central stimulus. We therefore tested the main effect of the Congruence for both ERs and RTs. In the continuity of these studies, we also compared congruence effects between central and peripheral vision through the interaction between the congruence and the distractor position. Concerning the main objectives of this experiment, we expected that the processing of the semantic information in the central ring was less biased by the selective attention on central fixation than the central disk. Therefore, we should observe a lower congruence effect from central vision on peripheral vision when the central stimulus is a ring than when it is a disk superimposed on the central fixation. To address this hypothesis, we thus tested for both ERs and RTs the interaction between the congruence and the eccentricity of the central stimulus for the peripheral target/central distractor task. Concerning the reverse task assessing the congruence effect from peripheral vision on central vision, we don't have a specific hypothesis on how categorizing a central ring rather than a central disk might be differently influenced by the presence of the peripheral distractor. However, for exploratory purposes, we also tested for both ERs and RTs the interaction between the Congruence and the Eccentricity of the central stimulus for the central target/peripheral distractor task.

### Results

**Figure 2.**
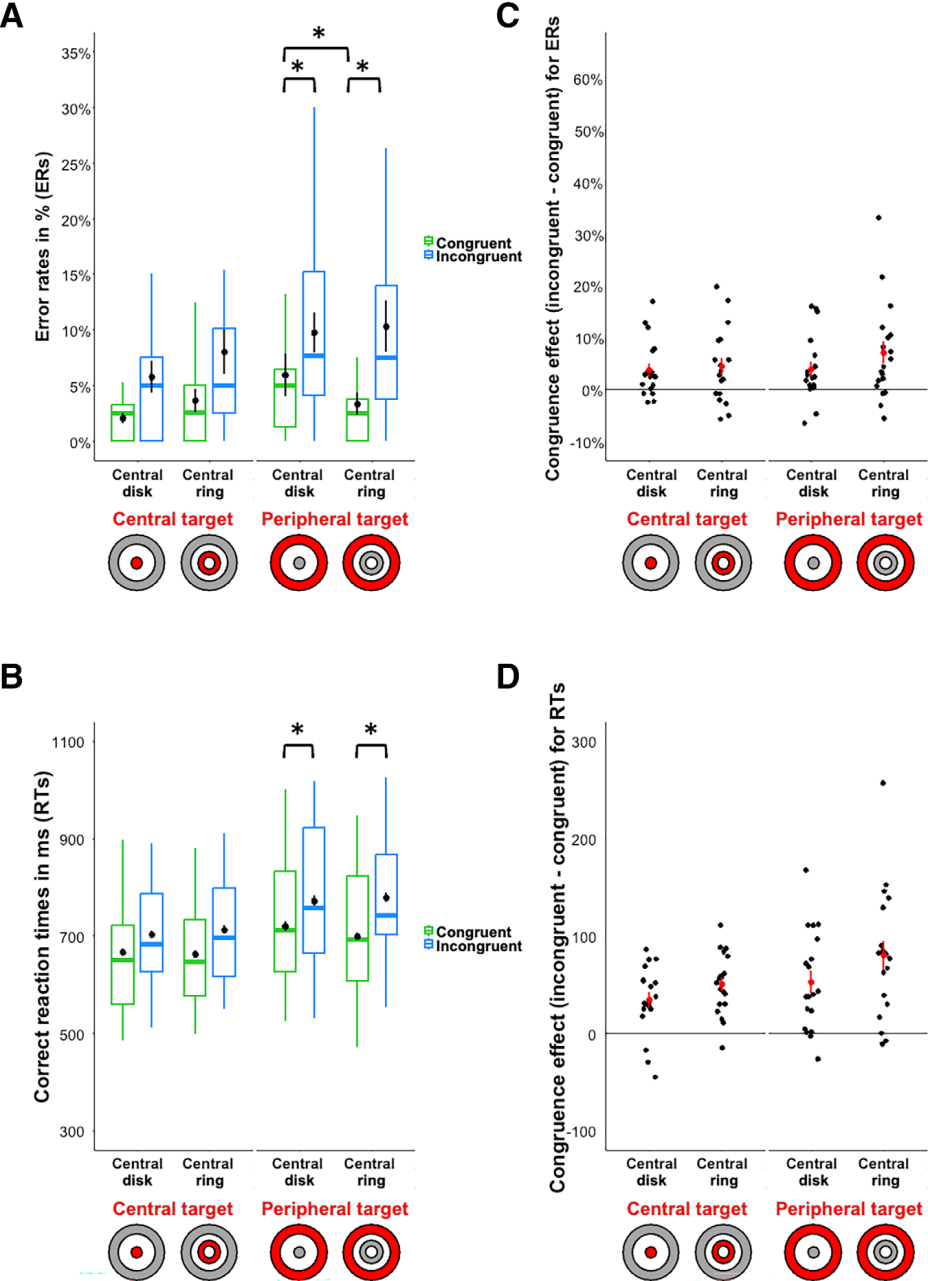
Box plots of (**A**) error rates in percentage, ERs and (**B**) correct response times in milliseconds (ms), RTs for categorizing the central or the peripheral target simultaneously displayed with a peripheral or a central distractor, respectively. The distractor is either semantically congruent (in green) or incongruent (in blue). Black dots indicate the mean and error bars indicate standard errors. Boxes represent medians and quartiles and whiskers represent the minimum and maximum sample without the outliers. Congruence effect (incongruent condition minus congruent condition) for (**C**) error rates in percentage and (**D**) correct response times in milliseconds (ms). Black dots indicate the mean congruence effect and error bars indicate standard errors. Red dots correspond to the congruence effect for each participant. Values above 0 indicate the presence of a congruent effect. Stimuli are illustrated on the x-axis: the target is in red and the distractor is in gray. The asterisk (*) means that the difference is significant (*p* < 0.05) for the comparisons tested.

Results are shown in Figure 2 and Table 1. The analyses performed on the ERs revealed a significant main effect of the Congruence, *β* = 0.96, *z* = 7.75, *p* < 0.001. Consistent with previous results (Lukavský, 2019; Trouilloud et al., 2022), participants made fewer errors to categorize the target stimulus when the two scenes were semantically congruent (mean ± standard error: 3.74% ± 0.35%) than incongruent (8.55% ± 0.52%). Also, we did not observe a significant interaction between the congruence and the Distractor position, *β* = −0.04, *z* = −0.17, *p* = 0.86. Concerning our main hypothesis, the interaction between the congruence and the Eccentricity of the central stimulus for the peripheral target/central distractor task was significant, *β* = 0.75, *z* = 2.40, *p* = 0.02. Planned comparison revealed a significant congruence effect whatever the position of the central stimulus (central disk: *β* = 0.83, *z* = 4.56, *p* < 0.001; central ring: *β* = 1.08, *z* = 6.36, *p* < 0.001). The significant interaction suggests thus an unexpected greater congruence effect from the central ring (congruent: 3.31% ± 0.66%; incongruent: 10.64% ± 1.15%) than from the central disk (congruent: 6.03% ± 0.88%; incongruent: 9.94% ± 1.11%) on the peripheral ring. In addition, we observed a significant effect of the eccentricity for semantically congruent trials only, *β* = −0.76, *z* = −2.22, *p* = 0.03 (semantically incongruent trials: *β* = −0.005, *z* = −0.19, *p* = 0.99). Participants made fewer errors to categorize the peripheral target when the central distractor was a semantically congruent ring (3.31% ± 0.66%) than when it was a disk (6.03% ± 0.88%). The interaction between the congruence and the eccentricity of the central stimulus was not significant for the central target/peripheral distractor condition, *β* = −0.24, *z* = −0.64, *p* = 0.52.

**Table 1.** Results of Experiment 1 for ERs and correct RTs. Significant results are in bold. *β* refers to the regression slope (i.e., the parameter of the fixed effect tested). The larger *β* is, the greater the slope, and the larger the effect. The final structure of the models used and how the predictors contrasts were coded are described in Supplementary Material (Tables A1 and A2).

	Statistics	
Effects	ERs	TRs
Congruence effect	***β* = 0.96**	***β* = 54.39**
	***z* = 7.75**	***t*(5436.74) = 10.74**
	***p* <** **0.001**	***p* <** **0.001, *d_z_* = 2.46**
Congruence × Distractor	*β* = −0.04	***β* = 23.39**
position	*z* = −0.17	***t*(5437.76) = 2.31**
	*p* = 0.86	***p* =** **0.02, *d_z_* = 0.53**
Planned comparisons		
Congruence for		***β* = 42.69**
peripheral distractor		***t*(5435.47) = 6.04**
		***p* <** **0.001, *d_z_* = 1.38**
Congruence for central		***β* = 66.08**
distractor		***t*(5433.38) = 9.10**
		***p* <** **0.001, *d_z_* = 2.09**
Distractor position for		***β* = 50.31**
congruent trial		***t*(22.10) = 3.20**
		***p* =** **0.004, *d_z_* = 0.73**
Distractor position for		***β* = 73.69**
incongruent trial		***t*(22.60) = 4.67**
		***p* <** **0.001, *d_z_* = 1.07**
Central distractor task only		
Congruence × Eccentricity	***β* = 0.75**	***β* = 29.61**
	***z* = 2.40**	***t*(5434.68) = 2.04**
	***p* =** **0.02**	***p* =** **0.04, *d_z_* = 0.47**
Planned comparisons		
Congruence for central	***β* = 0.83**	***β* = 43.26**
disk	***z* = 4.56**	***t*(5431.58) = 6.04**
	***p* <** **0.001**	***p* <** **0.001, *d_z_* = 1.39**
Congruence for central	***β* = 1.08**	***β* = 65.50**
ring	***z* = 6.36**	***t*(5434.41) = 9.13**
	***p* <** **0.001**	***p* <** **0.001, *d_z_* = 2.09**
Eccentricity for	***β* =** **−0.76**	*β* = −14.12
congruent trial	***z* =** **−2.22**	*t*(20.76) = −0.77
	***p* =** **0.03**	*p* = 0.45, *d_z_* = −0.18
Eccentricity for	*β* = −0.005	*β* = 8.06
incongruent trials	*z* = −0.19	*t*(21.11) = 0.44
	*p* = 0.99	*p* = 0.67, *d_z_* = 0.10
Peripheral distractor task only		
Congruence × Eccentricity	*β* = −0.24	*β* = 14.77
	*z* = −0.64	*t*(5435.66) = 1.05
	*p* = 0.52	*p* = 0.30, *d_z_* = 0.24

The analyses performed on RTs revealed also a significant main effect of the congruence, *β* = 54.39, *t*(5436.74) = 10.74, *p* < 0.001, *d_z_* = 2.46. Participants were faster to categorize the target stimulus when the two scenes were semantically congruent (686 ± 4 ms) than incongruent (740 ± 4 ms). For RTs, we observed this time a significant interaction between the congruence and the distractor position, *β* = 23.393, *t*(5437.76) = 2.31, *p* = 0.02, *d_z_* = 0.53. The congruence effect was significant for both distractor position conditions (peripheral distractor: *β* = 42.69, *t*(5435.47) = 6.04, *p* < 0.001, *d_z_* = 1.38; central distractor: *β* = 66.08, *t*(5433.38) = 9.10, *p* < 0.001, *d_z_* = 2.09). In addition, participants were faster for the central than peripheral target for both congruent stimuli, *β* = 50.31, *t*(22.10) = 3.20, *p* = 0.004, *d_z_* = 0.73, and incongruent stimuli, *β* = 73.69, *t*(22.60) = 4.67, *p* < 0.001, *d_z_* = 1.07). The significant interaction suggests thus a greater congruence effect from the central vision on the peripheral vision (congruent: 709 ± 6 ms; incongruent: 775 ± 7 ms) than from the peripheral vision on central vision (congruent: 664 ± 5 ms; incongruent: 708 ± 6 ms). Concerning our main hypothesis, as for the ERs, the interaction between the congruence and the eccentricity of the central stimulus was significant for the peripheral target/central distractor task, *β* = 29.61, *t*(5434.68) = 2.04, *p* = 0.04, *d_z_* = 0.47. Planned comparison revealed a significant effect of the congruence whatever the eccentricity of the central stimulus (central disk: *β* = 43.26, *t*(5431.58) = 6.04, *p* < 0.001, *d_z_* = 1.39; central ring: *β* = 65.50, *t*(5434.41) = 9.13, *p* < 0.001, *d_z_* = 2.09). The significant interaction suggests thus a greater congruence effect from the central ring (congruent: 699 ± 8 ms; incongruent: 779 ± 10 ms) than from the central disk (congruent: 720 ± 9 ms; incongruent: 772 ± 10 ms) on the peripheral ring. The eccentricity effect was not significant either for congruent trials, *β* = −14.12, *t*(20.76) = −0.77, *p* = 0.45, *d_z_* = −0.18, or incongruent trials, *β* = 8.06, *t*(21.11) = 0.44, *p* = 0.67, *d_z_* = 0.10. Finally, the interaction between the congruence and the eccentricity of the central stimulus was not significant for the central target/peripheral distractor condition, *β* = 14.77, *t*(5435.66) = 1.05, *p* = 0.30, *d_z_* = 0.24.

### Discussion of experiment 1

The purpose of this first experiment was to control for a potential experimental bias induced by a central fixation instruction in previous studies (Lukavský, 2019; Trouilloud et al., 2022) investigating congruence effects between central and peripheral vision. As a central stimulus, we used either a small central disk superimposed on the central fixation or a central ring slightly distant from the central fixation. The central fixation of participants was controlled by eye measurements. Only trials in which participants' fixation fell into the central disk area throughout the whole trial were retained. We expected that the congruence effect of the central stimulus on the peripheral stimulus would be lower when the central stimulus is distant than superimposed on the central fixation. We did observe a modulation of the congruence effect by this experimental manipulation, but contrary to our expectations, the congruence effect of the central stimulus was greater when it was not superimposed on the central fixation. This effect was observed for both error rates and reaction times. These unexpected results could be due to the particular design of the stimuli. We constructed our stimuli so that the surface of visual information revealed by each stimulus took into account the cortical magnification factor. Central and peripheral stimuli were designed to activate approximately the same quantity of neurons in the primary visual cortex. The counterpart is that the surface of a scene revealed by a stimulus increased with its eccentricity and the central ring potentially contained more useful information for categorization than the central disk. However, if the size of the congruence effect was determined by the surface of the distractor, the peripheral ring, which is the stimulus that contains the most scene information, should have induced the greatest congruence effect, and it was not the case.

Concomitantly with the increase in surface area for the central ring (compared to the central disk), it is also closer to the peripheral ring. Our results may then indicate that the congruence effect of central vision is stronger when the distance between the target and the distractor is the shortest. One possible candidate for such a mechanism are the long-range intra-cortical connections that are observed in the early visual cortex (Gilbert, Hirsch, & Wiesel, 1990) and which are notably involved in the extraction of border ownership (Zhaoping, 2005). However, the fact that there was no effect of the eccentricity of the central stimuli for central targets suggests that lateral connections act from central to periphery only making this alternative interpretation unconvincing. It should also be noted that participants made fewer errors to categorize the peripheral target when the central distractor was a semantically congruent ring than when it was a disk, while there was no significant difference in the incongruent condition. Therefore differences in congruence effect between the central disk and the central ring are mainly observed when stimuli are extracted from the same scene image. When looking at the stimuli, one can easily identify a continuity between the central ring and the peripheral ring (e.g., in Figure 1, the concrete lane divider that appears in the central ring continues at the bottom left of the peripheral ring). Although it also exists, this continuity is less evident between the central disk and the peripheral ring. The impression of continuity would lead to the perception of a single and unique stimulus in the visual field, and the central ring could finally be more difficult to ignore than the central disk. An experimental way to test this interpretation would be to construct semantically congruent compound stimuli by extracting central and peripheral stimuli from different scene images. However, although this would allow us to suppress the continuity bias between stimuli, it would not resolve the distance bias between the central and peripheral stimuli (i.e. the central ring would still be closer to the peripheral ring). Importantly, results of Experiment 1 demonstrated that central-peripheral visual interactions are in fact influenced by the distance between stimuli. The distance between different parts of the visual field is a physical property to be considered when investigating the interactions between central and peripheral vision. Overall, results of Experiment 1 argue against the idea that the congruence effect from central vision in peripheral categorization observed in previous studies (Lukavský, 2019; Trouilloud et al., 2022) could be explained by an increased selective attention at fixation.

## Experiment 2

In Experiment 2, we addressed the theoretical hypothesis that the interaction between central and peripheral vision relies on predictive top-down processes: According to predictive models of visual recognition, the rapid processing of LSF is used to trigger cortical predictive mechanisms that would then guide a more detailed visual analysis of HSF. We tested the hypothesis that the congruence effects between central and peripheral vision rely on the rapid processing of LSF by manipulating the spatial frequency content (LSF and HSF) of distractors. As this experiment was conducted in parallel with Experiment 1, we only used a central disk and a peripheral ring as stimuli as in the original paradigm (Lukavsky, 2019; Trouilloud et al., 2022). It should be noted that this also limits the possibility that the effects are somewhat due to perceptual continuity between central and peripheral stimuli.

### Method

#### Participants

Twenty undergraduate students of Psychology from University Grenoble Alpes (20 women; *M* ± *SD* = 19.27 ± 1.39 years) participated in this second experiment. The sample size choice was identically made as in Experiment 1, as well as the inclusion criteria and the ethical procedure.

#### Stimuli

**Figure 3.**
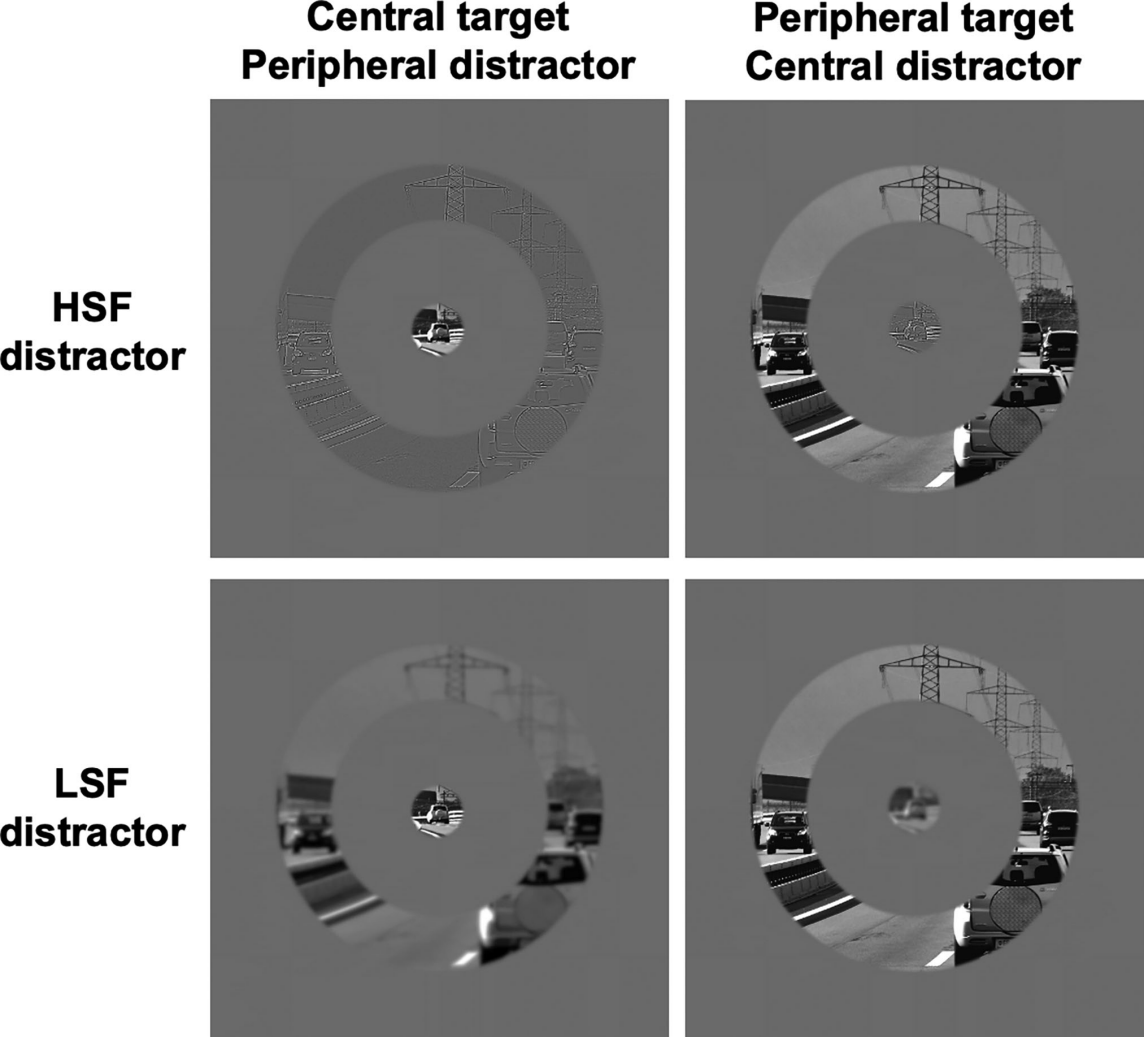
Example of congruent stimuli displayed in Experiment 2. A central disk and a peripheral ring extracted from the same scene were presented simultaneously. The distractor (either in central or peripheral position) was filtered either in HSF or LSF. It should be noted that the perception of spatial frequencies could be affected by the reduction of stimuli for an illustrative purpose.

The stimuli database used in Experiment 2 was exactly the same as in Experiment 1. Fifty scene images (25 outdoor images and 25 indoor images) were used and normalized to a mean luminance of 128 pixels intensity on a 256 gray level scale, and a mean Root Mean Square contrast of 51 on a 256 gray level scale. Using MATLAB (MathWorks Inc.), we filtered each image to obtain LSF and HSF scenes. For LSF scenes (Figure 3), we retained spatial frequencies below 2 cycles per degree (corresponding to 60 cycles per image). For HSF scenes, we retained spatial frequencies above 6 cycles per degree (corresponding to 180 cycles per image). The luminance contrast was not equalized between LSF and HSF stimuli as this type of experimental manipulation can impair the intrinsic properties of spatial filtered stimuli (Kauffman, Chauvin, Guyader, & Peyrin, 2015). Then, from nonfiltered and filtered images, we extracted a central disk and a peripheral ring. In total we had 150 central disks (50 nonfiltered, 50 filtered in LSF, and 50 filtered in HSF) and 150 peripheral rings (50 nonfiltered, 50 filtered in LSF, and 50 filtered in HSF). During the experiment, a central disk and a peripheral ring were simultaneously displayed. Depending on the experimental session, we displayed simultaneously either a central nonfiltered stimulus and a peripheral filtered stimulus (LSF or HSF filtered) or a peripheral nonfiltered stimulus and a central filtered stimulus (LSF or HSF filtered). This compound stimuli could be either congruent (e.g., an indoor scene in the central stimulus with the same indoor scene in the peripheral stimulus) or incongruent (e.g., an indoor scene in the central stimulus with an outdoor scene in the peripheral stimulus). Stimuli can be downloaded from https://osf.io/tez92/.

#### Procedure

Stimuli were displayed under the same conditions as in Experiment 1. All participants performed four experimental sessions. In two experimental sessions, we assessed the influence of the spatial content from peripheral vision on central vision. Participants had to categorize the nonfiltered central target stimulus (as belonging to the indoor or outdoor category), while ignoring the filtered peripheral distractor stimulus (central target/peripheral distractor task). The peripheral distractor stimulus was filtered in LSF in one session and in HSF in the other session. In the two other experimental sessions, we assessed the influence of the spatial content of central vision on peripheral vision. Participants had to categorize the nonfiltered peripheral target stimulus (as belonging to the indoor or outdoor category), while ignoring the filtered central distractor stimulus (peripheral target/central distractor task). The central distractor stimulus was filtered in LSF in one session and in HSF in the other session. The order of the four experimental sessions was counterbalanced between participants. As in Experiment 1, each trial began with a central fixation point for 500 ms, followed by a compound stimulus (either a nonfiltered central disk and a filtered peripheral ring or a nonfiltered peripheral ring and a filtered central disk) displayed for 100 ms. We presented stimuli for 100 ms to ensure that LSF and HSF information would be accurately processed in central vision. Indeed, Kauffmann et al. (2017) have shown that presentation times influence how spatial frequencies are processed in the way that HSF were not accurately processed for short presentation times (30 ms) whereas both LSF and HSF were accurately processed for longer presentation times (100 ms). In the same way as Experiment 1, participants had to categorize the target (either the central or peripheral stimulus) as an indoor or an outdoor scene, and we recorded eye movements throughout the experiment.

Each experimental session included 80 randomized trials (20 congruent compound stimuli belonging to the indoor category, 20 congruent compound stimuli belonging to the outdoor category, and 40 incongruent compound stimuli). Thus the whole experiment included 320 trials and lasted around 45 minutes. For each trial, we recorded the response accuracy and the response time in milliseconds (ms). Before starting each session, participants performed a training session of 20 trials with stimuli that were not included in the main experiment.

#### Data analysis

Data analyses were performed using the same methodology as in Experiment 1. For the error rates (ERs) analysis, we performed a generalized linear mixed-effects model (binomial family). Participants’ incorrect responses were coded 1 and correct responses were coded 0. For the correct response times (RTs) analyses, we performed a linear-mixed effects model of the congruence of the distractor (congruent vs. incongruent), the distractor position (peripheral distractor vs. central distractor), and the spatial frequency content of the distractor (LSF vs. HSF) in a 2 × 2 × 2 within-subjects design. For each model we included participants as a random factor. With the objective to generalize our results to other participants, we specified as random effects the intercepts for participants, along with participants-wise random slopes for congruence, distractor position, spatial frequency content of the distractor, and their mutual interactions. Data from two participants who did not perform the four experimental sessions were removed from the analyses. We also removed data from 2 participants who did not respect the central fixation instruction (57% of trials for one participant, and 91% of the trials for the other participant). Therefore we conducted the statistical analyses on 16 participants (15 women; *M* ± *SD* = 19.13 ± 0.96 years). For them, we removed 3.48% of the trials with incorrect fixation location.

Regarding previous studies (Lukavský, 2019; Trouilloud et al., 2022) and our results from Experiment 1, we expected to observe a main effect of the congruence, irrespective of the target/distractor position and of the spatial content of the distractor. Thus we tested the main effect of the congruence for both ERs and RTs. Furthermore, if congruence effects only result from the rapid extraction of LSF, we should observe a greater congruence effect from LSF than HSF distractor irrespective of the distractor position. To address this hypothesis, we tested for both ERs and RTs, the interaction between the congruence and the spatial frequency content of the distractor. If significant, we planned to test the congruence effect for each spatial frequency content of the distractor. Alternatively, if congruence effects result from the retinotopic characteristics of the visual system for processing spatial frequencies, we should observe a congruence effect from a central HSF distractor and a peripheral LSF distractor only. To address this alternative hypothesis, we thus tested for both ERs and RTs the three-way interaction between the congruence of the distractor, the distractor position, and the spatial frequency content of the distractor. If significant, we planned to test the Congruence effect for each Spatial frequency content of the distractor in each Distractor position.

### Results

**Figure 4.**
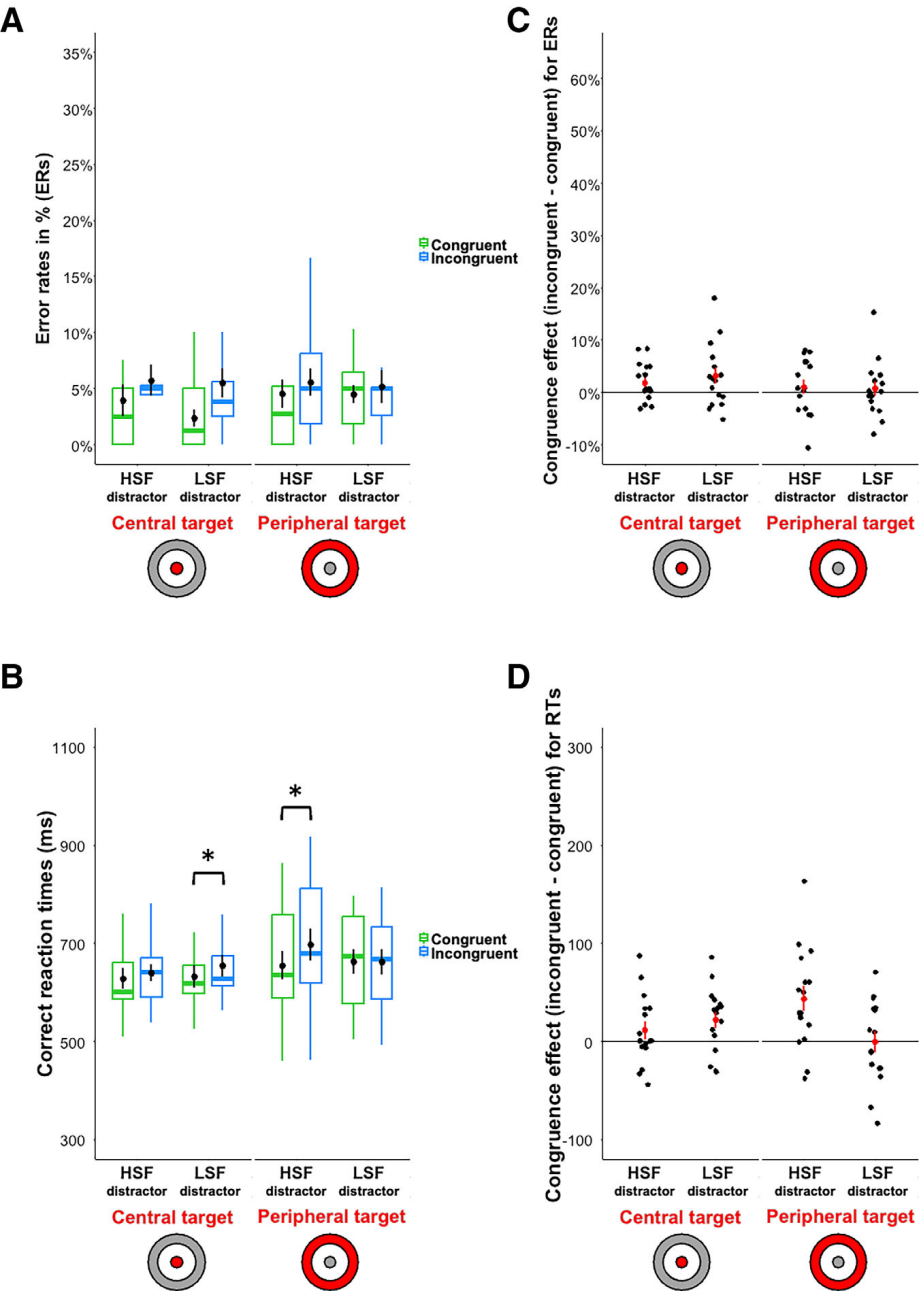
Box plots of (**A**) error rates in percentage, ERs and (**B**) correct response times in milliseconds (ms), RTs for categorizing the central or the peripheral target simultaneously displayed with a peripheral or a central distractor, respectively. The distractor is either semantically congruent (in green) or incongruent (in blue) and it is either filtered in LSF or HSF. Black dots indicate the mean and error bars indicate standard errors. Boxes represent medians and quartiles and whiskers represent the minimum and maximum sample without the outliers. Congruence effect (incongruent condition minus congruent condition) for (**C**) error rates in percentage and (**D**) correct response times in milliseconds (ms). Black dots indicate the mean congruence effect and error bars indicate standard errors. Red dots correspond to the congruence effect for each participant. Stimuli are illustrated on the x-axis: the target is in red and the distractor is in gray. The asterisk means that the difference tested is significant (*p* < 0.05) for the comparisons tested.

Results are shown in Figure 4 and Table 2. The analyses performed on ERs revealed a main effect of the Congruence, *β* = 0.43, *z* = 3.12, *p* < 0.001. As in Experiment 1 and previous studies (Lukavský, 2019; Trouilloud et al., 2022), participants made fewer errors to categorize the target stimulus when the two scenes were semantically congruent (3.73 ± 0.4%) than incongruent (5.50 ± 0.5%). Concerning our hypothesis, we did not observe an interaction neither between the congruence and the spatial frequency content of the distractor, *β* = −0.18, *z* = −0.63, *p* = 0.53, nor between the congruence of the distractor, the distractor position, and the spatial frequency content of the distractor, *β* = 0.65, *z* = 1.16, *p* = 0.25.

**Table 2.** Results of Experiment 2 for ERs and correct RTs. Significant results are in bold. *β* refers to the regression slope (i.e., the parameter of the fixed effect tested). The larger *β* is, the greater the slope and the larger the effect. The final structure of the models used and how the predictors contrasts were coded are described in Supplementary Material (Supplementary Table S3).

Effects	ERs	TRs
Congruence effect	***β* = 0.43**	***β* = 20.59**
	***z* = 3.12**	***t*(4642.28) = 4.18**
	***p* <** **0.001**	***p* <** **0.001, *d_z_* = 1.05**
Congruence × Spatial	*β* = −0.18	*β* = 17.80
frequency	*z* = −0.63	*t*(4641.76) = 1.81
	*p* = 0.53	*p* = 0.07, *d_z_* = 0.45
Congruence × Distractor ×	*β* = 0.65	***β* = 56.39**
Spatial frequency	*z* = 1.16	***t*(4642.02) =2.86**
	*p* = 0.25	***p* =** **0.004, *d_z_* = 0.72**
Planned comparisons		
Congruence for LSF		***β* = 22.25**
peripheral distractor		***t*(4642.23) = 2.30**
		***p* =** **0.02, *d_z_* = 0.58**
Congruence for HSF		*β* = 11.85
peripheral distractor		*t*(4642.11) =1.21
		*p* = 0.23, *d_z_* = 0.30
Congruence for LSF central		*β* = 1.14
distractor		*t*(4341.78) = 0.11
		*p* = 0.91, d_z_ = 0.03
Congruence for HSF central		***β* = 47.130**
distractor		***t*(4642.07) = 4.73**
		***p* <** **0.001, *d_z_* = 1.18**

The analyses performed on the RTs showed a main effect of the congruence, *β* = 20.59, *t*(4642.28) = 4.18, *p* < 0.001, *d_z_* = 1.05. Participants were faster to categorize the target stimulus when the two scenes were semantically congruent (640 ± 4 ms) than incongruent 662 ± 4 ms). Concerning our hypotheses, we did not observe a significant interaction between the congruence and the spatial frequency content of the distractor, *β* = 17.80, *t*(4641.76) = 1.81, *p* = 0.07, *d_z_* = 0.45. However, we observed a significant three-way interaction between the congruence, the target/distractor position, and the spatial frequency content of the distractor, *β* = 56.39, *t*(4642.02) =2.86, *p* = 0.004, *d_z_* = 0.72. The congruence effect was significant only when the peripheral distractor was filtered in LSF in the central target/peripheral distractor task (congruent: 630 ± 7 ms; incongruent: 655 ± 7 ms), *β* = 22.25, *t*(4642.23) = 2.30, *p* = 0.02, *d_z_* = 0.58, and when the central distractor was filtered in HSF in the peripheral target/central distractor task (congruent: 648 ± 8 ms; incongruent: 696 ± 10 ms), *β* = 47.130, *t*(4642.07) = 4.73, *p* < 0.001, *d_z_* = 1.18 (peripheral HSF distractor: *β* = 11.85, *t*(4642.11) =1.21, *p* = 0.23, *d_z_* = 0.30; central LSF distractor: *β* = 1.14, *t*(4341.78) = 0.11, *p* = 0.91, d_z_ = 0.03).

### Discussion of experiment 2

Results of Experiment 2 demonstrated that the spatial frequency content of distractors influences central-peripheral visual interactions: congruence effect of central vision on peripheral vision was only observed when the distractor contained HSF whereas congruence effect of peripheral vision on central vision was only observed when the distractor contained LSF. These results are consistent with the retinotopic organization of spatial frequency bottom-up processing from the retina to the visual cortex (Musel et al., 2013; Henriksson et al., 2008; Ramanoël et al., 2015; Sasaki et al., 2001; Singh et al., 2000). At the level of the retina, the density of cones and midget ganglion cells is the greatest in the fovea (Curcio & Allen, 1990; Curcio et al., 1990; Wässle et al., 1990). Therefore the central part of the retina is more suited to the processing of HSF information. With retinal eccentricity, the density of rods and parasol ganglion cells increases. As the receptive field of these cells are too large to extract HSF efficiently, the peripheral part of the retina is mainly sensitive to LSF. This functional organization is preserved at the level of the visual cortex. Neuroimaging studies demonstrated that the processing of HSF stimuli activated occipital cortical areas in relation to the representation of the fovea, whereas the processing of LSF stimuli activated other occipital areas in relation to the representation of a more peripheral part of the retina (Henriksson et al., 2008; Musel et al., 2013; Ramanoël et al., 2015). Therefore the congruence effect of central HSF on peripheral vision is consistent with the predominant bottom-up processing of HSF in central vision while the congruence effect of peripheral LSF on central vision is consistent with the predominant bottom-up processing of LSF in peripheral vision.

These results support the hypothesis that interactions between central and peripheral vision rely on the bottom-up retinotopic processing of spatial frequencies: LSF are mainly used in peripheral vision to be integrated to the central vision while HSF are mainly used in central vision to be integrated to the peripheral vision. Thus, contrary to our main hypothesis, the fact that previous studies (Lukavsky, 2019; Trouilloud et al., 2022) observed congruence effects of central vision as strong as congruence effects of peripheral vision cannot be explained by a rapid processing of LSF originating from both central and peripheral vision. However, the involvement of top-down mechanisms based on the rapid processing of LSF cannot be ruled out in peripheral vision. Indeed, LSF in peripheral vision would be used to trigger cortical predictive mechanisms that would then guide a more detailed visual analysis in central vision, resulting also in a congruence effect of peripheral LSF on central vision. Unfortunately, Experiment 2 did not allow us to conclude about how the two sources of information are integrated (i.e. whether one influences the other in a bottom-up or top-down manner). If the LSF peripheral influence is exerted in a top-down manner, semantically congruent LSF information should facilitate the categorization in central vision relative to its absence.

Experiment 3 was conducted to specifically investigate whether semantically congruent distractors caused facilitation by comparing performance between the congruent condition and a neutral condition in which the distractor was a 1/f noise without semantic content. We also compared performance between the incongruent condition and the neutral condition.

## Experiment 3

### Method

#### Participants

Eighteen undergraduate students of psychology from University Grenoble Alpes (17 women; *M* ± *SD* = 20.55 ± 2.04 years) participated in this third experiment. The sample size choice was identically made as in Experiments 1 and 2, as well as the inclusion criteria and the ethical procedure.

#### Stimuli and procedure

**Figure 5.**
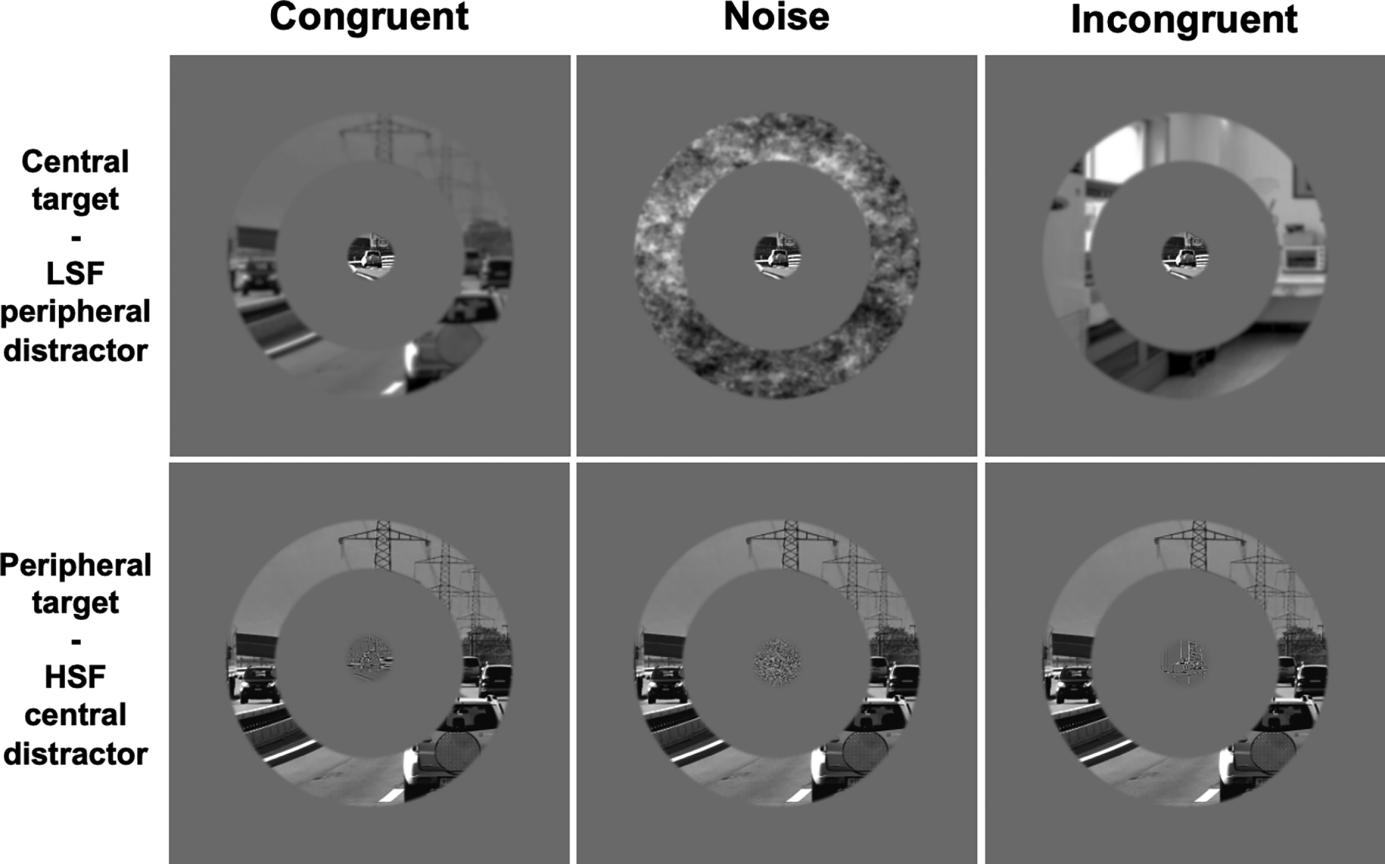
Example of stimuli displayed in Experiment 3. The central disk and the peripheral ring were presented simultaneously. When participants had to categorize the central disk (upper row), the peripheral ring distractor was filtered in LSF. It could be either a noise pattern, a semantically congruent scene or a semantically incongruent scene. When participants had to categorize the peripheral ring (lower row), the central disk distractor was filtered in HSF. It could be either a noise pattern, a semantically congruent scene or a semantically incongruent scene. It should be noted that the perception of spatial frequencies could be affected by the reduction of stimuli for an illustrative purpose.

The stimuli database used in Experiment 3 was exactly the same as in Experiment 2: Fifty scene images (25 outdoor images and 25 indoor images) either non filtered or filtered in LSF and HSF. We extracted from these stimuli a central disk and a peripheral ring as in Experiment 2. We also built an image with 1/f noise using MATLAB (MathWorks Inc.). This image was then filtered in LSF and HSF. From the HSF image, we extracted a central disk stimulus and from the LSF image, we extracted a peripheral ring (Figure 5). Noise images can be downloaded from https://osf.io/7qx8k/. Stimuli were displayed under the same conditions as Experiments 1 and 2.

As results of Experiment 2 demonstrated only a congruence effect of the central disk distractor when filtered in HSF and, on the contrary, only a congruence effect of the peripheral ring distractor when filtered in LSF, we only tested the influence of a HSF central disk and of a LSF peripheral ring. Therefore, to reduce the experiment duration, all participants performed four experimental sessions. In two experimental sessions, they had to categorize the nonfiltered central target stimulus (as belonging to the indoor or outdoor category), while ignoring the LSF peripheral distractor stimulus (central target/peripheral distractor task). In one of these two sessions, the LSF peripheral distractor stimulus contained semantic information (either congruent or incongruent), as in Experiment 2. In the other session, the LSF peripheral distractor was the noise stimulus without semantic information. In the two other experimental sessions, participants had to categorize the nonfiltered peripheral target stimulus (as belonging to the indoor or outdoor category), while ignoring the HSF central distractor stimulus (peripheral target/central distractor task). In one of these two sessions, the HSF central distractor stimulus contained semantic information (either congruent or incongruent), as in Experiment 2. In the other session, the HSF central distractor was the noise stimulus without semantic information.

As in Experiment 2, each trial began with a central fixation point, followed by a compound stimulus (either a nonfiltered central disk and a LSF filtered peripheral ring or a nonfiltered peripheral ring and a HSF filtered central disk) displayed for 100 ms. Participants had to categorize the target (either the central or peripheral stimulus) as an indoor or an outdoor scene and eye movements were recorded.

Each experimental session included 80 randomized trials (20 congruent compound stimuli belonging to the indoor category, 20 congruent compound stimuli belonging to the outdoor category, and 40 incongruent compound stimuli; or 80 compound stimuli with a noise as distractor). Thus, the whole experiment included 320 trials and lasted around 45 minutes. For each trial, we recorded the response accuracy and the response time in milliseconds (ms). Before starting each session, participants performed a training session of 20 trials with stimuli that were not included in the main experiment.

#### Data analysis

Data analyses were performed using the same methodology as in Experiments 1 and 2. For the error rates (ERs) analysis, we performed a generalized linear mixed-effects model (binomial family). Participants’ incorrect responses were coded 1 and correct responses were coded 0. For the correct response times (RTs) analyses, we performed a linear-mixed effects model of the Type of distractor (congruent vs. incongruent vs. noise) and the Distractor position (peripheral LSF distractor vs. central HSF distractor) in a 2 × 3 within-subjects design. For each model, we included participants as a random factor. With the objective to generalize our results to other participants, we specified as random effects the intercepts for participants, along with participants-wise random slopes for distractor position, type of distractor, and their mutual interactions. We removed 5.70% of the trials with incorrect fixation location.

Based on previous studies (Lukavský, 2019; Trouilloud et al., 2022) and results from Experiments 1 and 2, we expected to observe a main congruence effect (i.e., impaired performance in the incongruent conditions relative to the congruent conditions). We therefore tested the effect of the congruence for both ERs and RTs. Concerning the main objectives of this experiment, if semantic information in distractors is integrated in a top-down manner, we expect to observe better performance when the distractor is semantically congruent with the Target than when it has no semantic information (noise). To address this hypothesis, we thus tested the main effect of the type of semantic distractor (congruent vs noise, incongruent vs noise) for each distractor position (peripheral LSF distractor vs. central HSF distractor).

### Results

**Figure 6.**
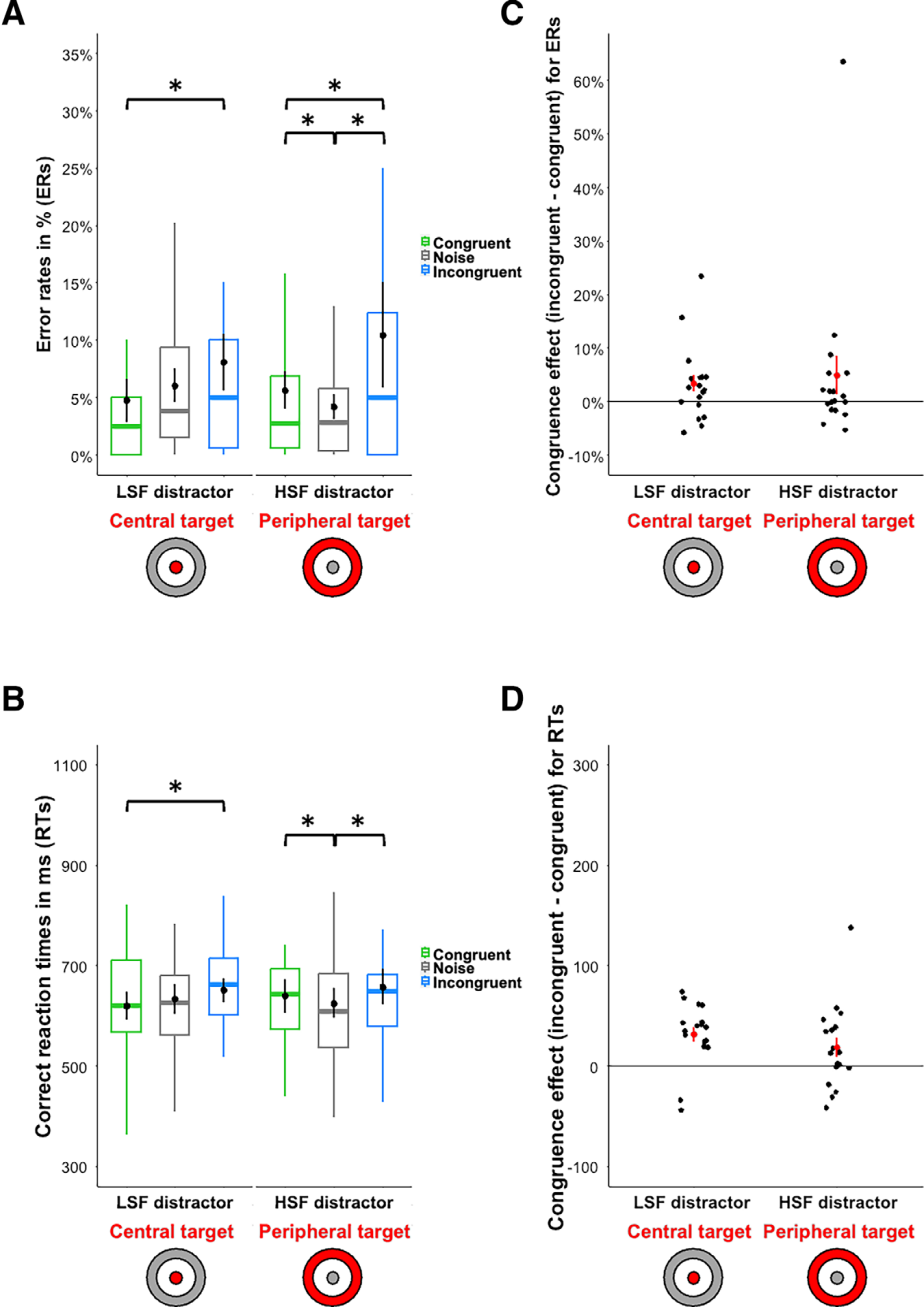
Box plots of (**A**) error rates in percentage, ERs and (**B**) correct response times in milliseconds (ms), RTs for categorizing the central or the peripheral target simultaneously displayed with a peripheral or a central distractor, respectively. The distractor is either semantically congruent (in green) or incongruent (in blue) or a noise pattern (in gray). The distractor was filtered in LSF when peripheral or HSF when central. Black dots indicate the mean and error bars indicate standard errors. Boxes represent medians and quartiles and whiskers represent the minimum and maximum sample without the outliers. Congruence effect (incongruent condition minus congruent condition) for (**C**) error rates in percentage and (**D**) correct response times in milliseconds (ms). Black dots indicate the mean congruence effect and error bars indicate standard errors. Red dots correspond to the congruence effect for each participant. Stimuli are illustrated on the x-axis: the target is in red and the distractor is in gray. The asterisk (*) means that the difference tested is significant (*p* < 0.05) for the comparisons tested.

Results are shown in Figure 6 and Table 3. As the manipulation of the spatial frequency content was different for the central target/peripheral LSF distractor task and for the peripheral target/central HSF distractor task, we conducted separate analyses for each task.

**Table 3.** Results of Experiment 3 for ERs and correct RTs. Significant results are in bold. *β* refers to the regression slope (i.e., the parameter of the fixed effect tested). The larger *β* is, the greater the slope and the larger the effect. The final structure of the models used and how the predictors contrasts were coded are described in Supplementary Material (Supplementary Table S4).

Effects	ERs	TRs
Central target/Peripheral distractor task	
Congruent vs. Incongruent	***β* = 0.61**	***β* = 30.43**
distractor	***z* = 2.73**	***t*(5044.07) = 3.34**
	***p* =** **0.006**	***p* <** **0.001, *d_z_* = 0.7**
Congruent vs. Noise	*β* = −0.12	*β* = 11.09
distractor	*z* = 0.63	t(5049.87) = 1.15
	*p* = 0.53	*p* = 0.15, *d_z_* = 0.34
Incongruent vs. Noise	*β* = 0.31	*β* = 10.89
distractor	*z* = −1.71	*t*(5051.42) = −1.40
	*p* = 0.09	*p* = 0.16, *d_z_* = −0.33
Peripheral target/Central distractor task	
Congruent vs. Incongruent	***β* = 0.80**	*β* = 11.88
distractor	***z* = 3.49**	t(5067.20) = 1.26
	***p* <** **0.001**	*p* = 0.21, *d_z_* = 0.30
Congruent vs. Noise	***β* =** **−0.41**	***β* =** **−19.82**
distractor	***z* =** **−2.18**	**t(5050.99) =** **−2.49**
	***p* =** **0.03**	***p* =** **0.01, *d_z_* =** **−0.59**
Incongruent vs. Noise	***β* =** **−0.96**	***β* =** **−29.26**
distractor	***z* =** **−5.12**	***t*(5050.53) =** **−3.63**
	***p* <** **0.001**	***p* <** **0.001, *d_z_* =** **−0.85**

For the central target/peripheral LSF distractor task, we observed a significant effect of the congruence on both ERs and RTs. Participants made fewer errors and they were faster to categorize the central target when the LSF peripheral distractor was semantically congruent (4.73% ± 0.81% and 625 ± 6 ms) than incongruent (8.29% ± 1.04% and 659 ± 7 ms), *β* = 0.61, *z* = 2.73, *p* = 0.006 and *β* = 30.43, *t*(5044.07) = 3.34, *p* < 0.001, *d_z_* = 0.78. Participants also made fewer errors and were faster to categorize the central disk when the LSF peripheral distractor was semantically congruent (4.73% ± 0.81% and 625 ± 6 ms) than when it was a noise pattern and therefore contained no semantic information (5.97% ± 0.63% and 642 ± 6 ms). However, this difference was not significant, *β* = −0.12, *z* = 0.63, *p* = 0.53 and *β* = 11.09, t(5049.87) = 1.15, *p* = 0.15, *d_z_* = 0.34, respectively. On the other hand, participants made more errors and were slower to categorize the central disk when the LSF peripheral distractor was semantically incongruent (8.29% ± 1.04% and 659 ± 7 ms) than when it was a noise pattern (5.97% ± 0.63% and 642 ± 6 ms). But, here again, this difference was not significant, *β* = 0.31, *z* = −1.71, *p* = 0.09 and *β* = 10.89, *t*(5051.42) = −1.40, *p* = 0.16, *d_z_* = −0.33, respectively.

For the peripheral target/central HSF distractor task, we only observed a significant effect of the congruence for the ERs. Participants made fewer errors to categorize the peripheral target when the HSF central distractor was semantically congruent (5.93% ± 0.91%) than incongruent (10.63 ± 1.20%), *β* = 0.80, *z* = 3.49, *p* < 0.001. But this difference was not significant for the RTs (Congruent: 646 ± 8 ms; Incongruent: 653 ± 8 ms), *β* = 11.88, t(5067.20) = 1.26, *p* = 0.21, *d_z_* = 0.30. Moreover, participants made fewer errors and were faster to categorize the peripheral target when the HSF central distractor was a noise pattern (4.36% ± 0.57%; 625 ± 6 ms) than when it was a distractor with semantic information, whether congruent, (5.93% ± 0.91%; 646 ± 8 ms), *β* = −0.41, *z* = −2.18, *p* = 0.03 and *β* = −19.82, t(5050.99) = −2.49, *p* = 0.01, *d_z_* = −0.59, or incongruent, (10.63 ± 1.20%, 653 ± 8 ms), *β* = −0.96, *z* = −5.12, *p* < 0.001 et *β* = −29.26, *t*(5050.53) = −3.63, *p* < 0.001, *d_z_* = −0.85.

## General discussion

Central and peripheral vision are processed interactively during scene categorization. Such interactions were previously investigated through the semantic congruence effect by Lukavský (2019) and Trouilloud et al. (2022). These studies revealed that participants’ performance for categorizing a part of a scene (either central or peripheral) was better when the central and the peripheral parts were congruent than incongruent. This congruence effect was observed whatever the location (central or peripheral) of the part to categorize, suggesting that the information to be ignored was automatically processed and integrated to the processing of the visual information to attend. In addition, these studies revealed a congruence effect of central vision on peripheral vision as strong as the reverse, suggesting a common integration mechanism. In the present study, we conducted three experiments to further understand the mechanisms underlying these interactions.

The general paradigm was to present simultaneously two stimuli of different retinal eccentricity, one central and one peripheral, that could be either semantically congruent (belonging to the same scene image) or incongruent (belonging to two different scene images from different categories). To assess the congruence effect of central vision on peripheral vision, participants had to categorize the peripheral target stimulus (as an indoor or an outdoor scene) while ignoring the central distractor stimulus and in order to assess the congruence effect of the peripheral vision on central vision, they had to categorize the central target stimulus while ignoring the peripheral distractor stimulus. Experiment 1 suggested that the physical distance between central and peripheral stimuli influences central-peripheral visual interactions: Congruence effect of central vision is stronger when the distance between the target and the distractor is the shortest. Thus, the distance between different parts of the visual field is a physical property to be considered when investigating the interactions between central and peripheral vision. Experiments 2 and 3 further revealed that central-peripheral visual interactions are also influenced by other physical properties of the visual information, such as the spatial frequency content.

Experiment 2 was originally theoretically motivated by the hypothesis that congruence effects observed between central and peripheral vision rely on predictive brain mechanisms. The rapid extraction and conduction of LSF from the retina to the brain would allow a global and rudimentary representation of an object or the scene that could be used to generate predictions about a more detailed representation in HSF. We therefore manipulated the spatial frequency content of distractors (either filtered in LSF or HSF) and we only used a central disk and a peripheral ring as stimuli as in previous studies (Lukavsky, 2019; Trouilloud et al., 2022). Participants were asked to categorize the target scene (either the central disk or the peripheral ring), and to ignore the filtered distractor (either the peripheral ring or the central disk, respectively). If congruence effects resulted from the rapid extraction of an LSF content that triggers top-down predictive processes, we expected to observe a greater congruence effect from LSF distractors than from HSF distractors (whether central or peripheral distractors). This hypothesis was, however, not supported by the results of Experiment 2. Congruence effects were differently influenced by the spatial frequency content of distractors depending on their position in the visual field. Indeed, we observed a congruence effect of the central distractor only when its content was filtered in HSF and a congruence effect of the peripheral distractor only when its content was filtered in LSF. In other words, we observed the influence of central HSF on peripheral vision and the influence of peripheral LSF on central vision. Results of Experiment 2 are consistent with the retinotopic organization of spatial frequency bottom-up processing from the retina to the visual cortex (Musel et al., 2013; Henriksson et al., 2008; Ramanoël et al., 2015; Sasaki et al., 2001; Singh et al., 2000): LSF are mainly used in peripheral vision to be integrated to the central vision whereas HSF are mainly used in central vision to be integrated to the peripheral vision. Thus, contrary to our main hypothesis, congruence effects as strong from central vision as from peripheral vision (Lukavsky, 2019; Trouilloud et al., 2022) cannot be explained by a rapid processing of LSF originating from both central and peripheral vision. However, the presence of concomitant top-down mechanisms based on the rapid processing of LSF cannot be ruled out in peripheral vision. This hypothesis is based on the functional properties of the spatial frequency processing and previous psychophysical and neuroimaging studies. LSF in a scene are conveyed by the fast magnocellular pathways from the retina to the cortex and would then reach more rapidly high level cortical areas than HSF conveyed more slowly by the parvocellular pathways (Bullier, 2001). Behavioral studies in humans confirmed that the LSF content is extracted faster than HSF (Breitmeyer, 1975; Kauffmann et al., 2017). Besides, consistent with the main extraction of LSF in peripheral vision, behavioral studies that investigated the relative contribution of central and peripheral vision for scene categorization systematically showed that the peripheral part of a scene is categorized faster than its central part (Larson & Loschky, 2009; Loschky et al., 2019; Trouilloud et al., 2020). Furthermore, predictive models of object and scene recognition (Bar, 2003; Bar, 2007; Kveraga et al., 2007; Kauffmann, Chauvin, Pichat et al., 2015; Oliva & Torralba, 2006; Peyrin et al., 2010) assume that the rapid extraction of LSF would guide the subsequent processing of HSF in a top-down manner. Using fMRI, Kauffmann, Chauvin, Pichat, et al. (2015) revealed that the LSF content in a scene increased the top-down connectivity from the orbitofrontal cortex to the inferior temporal cortex to influence the bottom-up processing of the HSF content. Peyrin et al. (2021) subsequently demonstrated that semantic and physical information in peripheral vision influence the categorization of scenes in central vision, involving the same cortical network. In this context, the congruence effect of LSF only observed in peripheral vision is consistent with the bottom-up retinotopic processing of LSF, and top-down mechanisms could act concomitantly: LSF information mainly extracted from the peripheral distractor could rapidly reach high-order areas allowing to generate predictions that would guide the subsequent processing of central information. If this is the case, the presence of congruent semantic LSF information in peripheral vision should facilitate the categorization compared to incongruent semantic LSF information (this is the congruent effect observed), but also compared to the absence of semantic information.

We conducted Experiment 3 to specifically investigate whether semantically congruent distractors caused facilitation by comparing performance between the congruent condition and a neutral condition in which the distractor was a 1/f noise without semantic content. We reproduced the congruence effect of the LSF peripheral ring on the categorization of the central disk for both accuracy and reaction times, but we only reproduced the congruence effect of the HSF central disk on the categorization of the peripheral ring disk for accuracy, this effect being no longer observed for reaction times. This discrepancy can be explained by two main factors. First, in Experiment 2, we investigated the congruence effect of the central distractor by testing both HSF and LSF central disks while, in Experiment 3, we only tested the HSF central disk. As the manipulation of spatial frequencies was applied in different sessions, we hardly assume that the absence of a condition with a LSF central disk as a distractor alone accounts for these differences. Alternatively, in Experiment 3, the introduction of an experimental condition without semantic information in distractors (even if counterbalanced with the other experimental condition) would ultimately have the effect of decreasing the experimental interest in processing the content of the central distractor and therefore of decreasing a potential selective attention bias on the central distractor investigated in Experiment 1.

Concerning our specific hypothesis of a facilitation by a LSF congruent distractor, results of Experiment 3 showed that performances (in accuracy and reaction times) were better when there was a congruent LSF distractor than when there was a noise (no semantic information), but differences failed to reach significance. It should be noted, however, that the absence of statistical significance could be due to a lack of statistical power because the sample size was chosen by performing a power analysis based on the congruence effect (i.e., the comparison between congruent and incongruent conditions) observed in Trouilloud et al., 2022 and not based on a facilitation effect (i.e., the comparison between congruent and noise conditions). On the contrary, results showed that performances (in accuracy and reaction times) for the Peripheral Target/Central HSF distractor task were this time significantly impaired when there was a congruent HSF distractor than when there was a noise. This pattern of results suggests different mechanisms behind the influence of LSF in peripheral vision vs. HSF in central vision. Unfortunately, we could not test the interaction between the Distractor position and the Spatial frequency content of the distractor. We were constrained to conduct separate statistical analyses for each task (as we manipulated concomitantly the position and the spatial frequency content of the distractor). Interestingly, if we summarize the results for the HSF central distractor (in comparison to the LSF peripheral distractor), the congruence effect is not systematically observed for reaction times and reaction times are longer for both semantically congruent and incongruent conditions than for the noise condition. This pattern of results could thus indicate that peripheral information alone is sufficient to quickly and accurately categorize the scene. When there is semantic information in HSF-central vision, it would however be additionally processed allowing to further accumulate evidence until the amount of information is enough to reach a decision on the category (Lukavský, 2019) and confirm periphery-based categorization. This could therefore result in slightly increasing reaction times but also uncertainty about the category leaving more room for errors when central semantic information is congruent to peripheral information than when no semantic information is available in central vision (no further accumulation of semantic information leading to faster and more straightforward categorization). When central information is semantically incongruent and conflicts with peripheral categorization, it would interfere with it leading to the congruence effect observed in terms of accuracy. Indeed, even if categorization can be performed based on peripheral information only, it may however be difficult and even counter-productive to ignore the evidence accumulated in HSF-central information, which may be more reliable to disentangle between alternative interpretations when it conflicts with coarse peripheral information (see also Kauffmann et al., 2017 for a similar rationale). To summarize results of Experiment 3, we propose that interactions between central and peripheral vision rely on the bottom-up retinotopic processing of spatial frequencies, although the presence of a top-down mechanism in peripheral vision cannot be totally ruled out. Future neuroimaging studies could be very useful to further investigate bottom-up and top-down processes underlying central-peripheral visual interactions.

In conclusion, these results go further than those observed in previous studies using very similar experimental paradigms (Lukavsky, 2019; Trouilloud et al., 2022). Previous studies showed that central and peripheral vision strongly interact during scene categorization, and that the influence of central vision on peripheral vision would be as strong as that of peripheral vision on central vision, suggesting that interaction between central and peripheral vision would not depend on the position of the information in the visual field. The present study suggests however different mechanisms behind the respective influences between central and peripheral vision that would strongly depend on the functional and anatomical properties of the processing of spatial frequencies from the retina to the cortex. It remains that in this study, the congruence was manipulated at the image level: congruent stimuli were built from the same scene as in natural conditions, while incongruent stimuli were built from different scenes. Therefore, we cannot conclude about congruence effects at the level of category processing. However, previous studies demonstrated congruence effects between central and peripheral vision even when the two sources of information were extracted from different images (Lukavský, 2019; Roux-Sibilon et al., 2019; Peyrin et al., 2021). We should expect central and peripheral interactions at the level of the category processing, although it has to be experimentally demonstrated in future studies.

## Supplementary Material

Supplement 1
