# Supplementary material for: Interaction between central and peripheral vision: Influence of distance and spatial frequencies
Source: J Vis. 2024 Jan 8;24(1):3. doi: 10.1167/jov.24.1.3 (PMC10777871; doi:10.1167/jov.24.1.3)
Supplement: Supplement 1 [file jovi-24-1-3_s001.pdf]

Effects	Contrasts
ERs ~ Congruence * Distractor position * Eccentricity of the central stimulus + (1 + Eccentricity + Distractor position : Eccentricity || participant)	
**Congruence effect**	**Congruence:** Congruent ← -0.5; Incongruent ← 0.5<br>**Distractor position:** Peripheral distractor ←-0.5; Central distractor ← 0.5<br>**Eccentricity:** Central disk ← -0.5; Central ring ← 0.5
**Congruence ╳ Distractor position**	
**Central distractor task only**	
**Congruence ╳ Eccentricity**	**Congruence:** Congruent ← -0.5; Incongruent ← 0.5<br>**Distractor position:** Peripheral distractor ←1; Central distractor ← 0<br>**Eccentricity:** Central disk ← -0.5; Central ring ← 0.5
**Planned comparisons**	
Congruence effect for central disk	**Congruence:** Congruent ← -0.5; Incongruent ← 0.5<br>**Distractor position:** Peripheral distractor ←1; Central distractor ← 0<br>**Eccentricity:** Central disk ← 0; Central ring ← 1
Congruence effect for central ring	**Congruence:** Congruent ← -0.5; Incongruent ← 0.5<br>**Distractor position:** Peripheral distractor ←1; Central distractor ← 0<br>**Eccentricity:** Central disk ← 1; Central ring ← 0
Eccentricity effect for congruent trials	**Congruence:** Congruent ← 0; Incongruent ← 1<br>**Distractor position:** Peripheral distractor ←1; Central distractor ← 0<br>**Eccentricity:** Central disk ← -0.5; Central ring ← 0.5
Eccentricity effect for incongruent trials	**Congruence:** Congruent ← 1; Incongruent ← 0<br>**Distractor position:** Peripheral distractor ←1; Central distractor ← 0<br>**Eccentricity:** Central disk ← -0.5; Central ring ← 0.5
**Peripheral distractor task only**	
**Congruence ╳ Eccentricity**	**Congruence:** Congruent ← -0.5; Incongruent ← 0.5<br>**Distractor position:** Peripheral distractor ←0; Central distractor ←1<br>**Eccentricity:** Central disk ← -0.5; Central ring ← 0.5

Table A1. Final generalized linear mixed-effect model (GLMM) for ERs in Experiment 1 with the fixed effects (in green in the model) and associated contrasts code. The random effect part, in blue in the model, indicates that the model estimates the intercept and slopes for the main effect of the Eccentricity of the central stimulus and for the interaction between the Distractor position and the Eccentricity of the central stimulus for each participant.

Effects	Contrasts
TRs ~ Congruence * Distractor position * Eccentricity of the central stimulus + (1 + Congruence + Distractor position + Eccentricity + Distractor position : Eccentricity || participant)	
**Congruence effect**<br>**Congruence ✕ Distractor position**	**Congruence:** Congruent 🡐 -0.5; Incongruent 🡐 0.5<br>**Distractor position:** Peripheral distractor 🡐-0.5; Central distractor 🡐 0.5<br>**Eccentricity:** Central disk 🡐 -0.5; Central ring 🡐 0.5
**Planned comparisons**	
Congruence for peripheral distractors	**Congruence:** Congruent 🡐 -0.5; Incongruent 🡐 0.5<br>**Distractor position:** Peripheral distractor 🡐 0; Central distractor 🡐 1<br>**Eccentricity:** Central disk 🡐 -0.5; Central ring 🡐 0.5
Congruence for central distractors	**Congruence:** Congruent 🡐 -0.5; Incongruent 🡐 0.5<br>**Distractor position:** Peripheral distractor 🡐 1; Central distractor 🡐 0<br>**Eccentricity:** Central disk 🡐 -0.5; Central ring 🡐 0.5
Distractor position for congruent trials	**Congruence:** Congruent 🡐 0; Incongruent 🡐 1<br>**Distractor position:** Peripheral distractor 🡐-0.5; Central distractor 🡐 0.5<br>**Eccentricity:** Central disk 🡐 -0.5; Central ring 🡐 0.5
Distractor position for incongruent trials	**Congruence:** Congruent 🡐 1; Incongruent 🡐 0<br>**Distractor position:** Peripheral distractor 🡐-0.5; Central distractor 🡐 0.5<br>**Eccentricity:** Central disk 🡐 -0.5; Central ring 🡐 0.5
**Central distractor task only**	
**Congruence ✕ Eccentricity**	**Congruence:** Congruent 🡐 -0.5; Incongruent 🡐 0.5<br>**Distractor position:** Peripheral distractor 🡐1; Central distractor 🡐 0<br>**Eccentricity:** Central disk 🡐 -0.5; Central ring 🡐 0.5
**Planned comparisons**	
Congruence effect for central disk	**Congruence:** Congruent 🡐 -0.5; Incongruent 🡐 0.5<br>**Distractor position:** Peripheral distractor 🡐1; Central distractor 🡐 0<br>**Eccentricity:** Central disk 🡐 0; Central ring 🡐 1
Congruence effect for central ring	**Congruence:** Congruent 🡐 -0.5; Incongruent 🡐 0.5<br>**Distractor position:** Peripheral distractor 🡐1; Central distractor 🡐 0<br>**Eccentricity:** Central disk 🡐 1; Central ring 🡐 0
Eccentricity effect for congruent trials	**Congruence:** Congruent 🡐 0; Incongruent 🡐 1<br>**Distractor position:** Peripheral distractor 🡐1; Central distractor 🡐 0<br>**Eccentricity:** Central disk 🡐 -0.5; Central ring 🡐 0.5
Eccentricity effect for incongruent trials	**Congruence:** Congruent 🡐 1; Incongruent 🡐 0<br>**Distractor position:** Peripheral distractor 🡐1; Central distractor 🡐 0<br>**Eccentricity:** Central disk 🡐 -0.5; Central ring 🡐 0.5
**Peripheral distractor task only**	
**Congruence ✕ Eccentricity**	**Congruence:** Congruent 🡐 -0.5; Incongruent 🡐 0.5<br>**Distractor position:** Peripheral distractor 🡐0; Central distractor 🡐1<br>**Eccentricity:** Central disk 🡐 -0.5; Central ring 🡐 0.5

Table A2. Final linear mixed-effect model (LMM) for RTs in Experiment 1 with the fixed effects (in green in the model) and associated contrasts code. The random effect part, in blue in the model, indicates that the model estimates the intercept and slopes for the main effect of the Congruence, the Distractor position, the Eccentricity of the central stimulus and for the interaction between the Distractor position and the Eccentricity of the central stimulus for each participant.

Effects	Contrasts
ERs ~ Congruence*Distractor position *Spatial frequency content of the distractor + (1 | participant)	
**Congruence effect**	**Congruence:** Congruent ← -0.5; Incongruent ← 0.5<br>**Distractor position:** Peripheral distractor ←-0.5; Central distractor ← 0.5<br>**Spatial frequency content:** LSF distractor ← -0.5; HSF distractor ← 0.5
**Congruence ✕ Spatial frequency**	
**Congruence ✕ Distractor position ✕ Spatial frequency**	
TRs ~ Congruence*Distractor position *Spatial frequency content of the distractor + (1 + Distractor position + Spatial frequency content of the distractor + Distractor position : Spatial frequency | participant)	
**Congruence effect**	**Congruence:** Congruent ← -0.5; Incongruent ← 0.5<br>**Distractor position:** Peripheral distractor ←-0.5; Central distractor ← 0.5<br>**Spatial frequency content:** LSF distractor ← -0.5; HSF distractor ← 0.5
**Congruence ✕ Spatial frequency**	
**Congruence ✕ Distractor position ✕ Spatial frequency**	
**Planned comparisons**	
Congruence effect for LSF peripheral distractor	**Congruence:** Congruent ← -0.5; Incongruent ← 0.5<br>**Distractor position:** Peripheral distractor ← 0; Central distractor ← 1<br>**Spatial frequency content:** Central LSF ← 0; HSF← 1
Congruence effect for HSF peripheral distractor	**Congruence:** Congruent ← -0.5; Incongruent ← 0.5<br>**Distractor position:** Peripheral distractor ← 0; Central distractor ← 1<br>**Spatial frequency content:** LSF distractor ← 1; HSF distractor ← 0
Congruence effect for LSF central distractor	**Congruence:** Congruent ← -0.5; Incongruent ← 0.5<br>**Distractor position:** Peripheral distractor ← 1; Central distractor ← 0<br>**Spatial frequency content:** LSF distractor ← 0; HSF distractor ← 1
Congruence effect for HSF central distractor	**Congruence:** Congruent ← -0.5; Incongruent ← 0.5<br>**Distractor position:** Peripheral distractor ← 1; Central distractor ← 0<br>**Spatial frequency content:** LSF distractor ← 1; HSF distractor ← 0

Table A3. Final generalized linear mixed-effect model (GLMM) for ERs and linear mixed-effect model (LMM) for RTs in Experiment 2 with the fixed effects (in green in the models) and associated contrasts code. The random effect part, in blue in the models, indicates that the GLMM estimates the intercept for each participant. The LMM estimates the intercept and slopes for the main effect of the Distractor position, the Spatial frequency content of the distractor, and the interaction between the Distractor position and the Spatial frequency content of the distractor for each participant.

Effects	Contrasts
ERs ~ Congruence + (Congruence || participant)	
**Central target/Peripheral distractor task**	
Congruent vs. Incongruent distractor	**Congruence:** Congruent ← -0.5; Incongruent ← 0.5; Noise ← 0
Congruent vs. Noise distractor	**Congruence:** Congruent ← -0.5; Incongruent ← 0; Noise ← 0.5
Incongruent vs. Noise distractor	**Congruence:** Congruent ← 0; Incongruent ← -0.5; Noise ← 0.5
**Peripheral target/Central distractor task**	
Congruent vs. Incongruent distractor	**Congruence:** Congruent ← -0.5; Incongruent ← 0.5; Noise ← 0
Congruent vs. Noise distractor	**Congruence:** Congruent ← -0.5; Incongruent ← 0; Noise ← 0.5
Incongruent vs. Noise distractor	**Congruence:** Congruent ← 0; Incongruent ← -0.5; Noise ← 0.5
TRs ~ Congruence + (Congruence || participant)	
**Central target/Peripheral distractor task**	
Congruent vs. Incongruent distractor	**Congruence:** Congruent ← -0.5; Incongruent ← 0.5; Noise ← 0
Congruent vs. Noise distractor	**Congruence:** Congruent ← -0.5; Incongruent ← 0; Noise ← 0.5
Incongruent vs. Noise distractor	**Congruence:** Congruent ← 0; Incongruent ← -0.5; Noise ← 0.5
**Peripheral target/Central distractor task**	
Congruent vs. Incongruent distractor	**Congruence:** Congruent ← -0.5; Incongruent ← 0.5; Noise ← 0
Congruent vs. Noise distractor	**Congruence:** Congruent ← -0.5; Incongruent ← 0; Noise ← 0.5
Incongruent vs. Noise distractor	**Congruence:** Congruent ← 0; Incongruent ← -0.5; Noise ← 0.5

Table A4. Final generalized linear mixed-effect model (GLMM) for ERs and linear mixed-effect model (LMM) for RTs in Experiment 3 with the fixed effects (in green in the model) and associated contrasts code. The random effect part, in blue in the models, indicates that the both models estimate the Congruence effect for each participant.
